# Intelligent Joint Space Path Planning: Enhancing Motion Feasibility with Goal-Driven and Potential Field Strategies

**DOI:** 10.3390/s25144370

**Published:** 2025-07-12

**Authors:** Yuzhou Li, Yefeng Yang, Kang Liu, Chih-Yung Wen

**Affiliations:** Department of Aeronautical and Aviation Engineering, The Hong Kong Polytechnic University, Hong Kong 999077, China; yuzhou5.li@connect.polyu.hk (Y.L.); yefeng.yang@connect.polyu.hk (Y.Y.); kang1.liu@polyu.edu.hk (K.L.)

**Keywords:** collision avoidance, path planning, manipulator, joint space, rapidly-exploring random tree

## Abstract

Traditional path-planning algorithms for robotic manipulators typically focus on end-effector planning, often neglecting complete collision avoidance for the entire manipulator. Additionally, many existing approaches suffer from high time complexity and are easily trapped in local extremes. To address these challenges, this paper proposes a goal-biased bidirectional artificial potential field-based rapidly-exploring random tree* (GBAPF-RRT*) algorithm, which enhances both target guidance and obstacle avoidance capabilities of the manipulator. Firstly, we utilize a Gaussian distribution to add heuristic guidance into the exploration of the robotic manipulator, thereby accelerating the search speed of the RRT*. Then, we combine the modified repulsion function to prevent the random tree from trapping in a local extreme. Finally, sufficient numerical simulations and physical experiments are conducted in the joint space to verify the effectiveness and superiority of the proposed algorithm. Comparative results indicate that our proposed method achieves a faster search speed and a shorter path in complex planning scenarios.

## 1. Introduction

In modern industrial manufacturing, automated production, medical surgery, aerospace, and precision assembly, robotic manipulators are widely utilized for various complex tasks due to their high precision, efficiency, and programmability [1,2,3]. These tasks include welding, material handling, spraying, assembly, and remote operations in hazardous environments. However, in such complex working environments, numerous obstacles may interfere with the operation of robotic manipulators, necessitating obstacle avoidance capabilities to ensure smooth task execution. Therefore, path-planning technology plays a crucial role in enabling manipulators to complete designated tasks safely and efficiently. Compared to low-degree-of-freedom (DOF) manipulators, high-DOF manipulators offer greater flexibility and optimization potential in path planning, especially in cluttered or constrained environments. However, efficiently planning motion paths for such manipulators remains a key research challenge in the field. The difficulty lies in achieving both obstacle avoidance and trajectory feasibility.

Path planning algorithms are typically classified into four major categories based on their computational approach and characteristics, namely, searching-based methods, sampling-based methods, optimization-based methods, and Reinforcement Learning-based methods.

The first category consists of searching-based methods, such as A* and Dijkstra. A* is a heuristic search algorithm that uses an evaluation function to guide the search direction and move toward the goal. Lin et al. proposed an improved A* algorithm that achieves shorter paths and fewer inflection points, thereby optimizing path quality. However, it introduces higher computational complexity, making it less scalable in high-dimensional spaces [4]. Dijkstra’s algorithm is another classic approach for finding the shortest path in a weighted map. Buzachis et al. modified the algorithm to optimize the path and leveraged MapReduce to improve computation speed [5]. Similarly, Prasad et al. designed a multi-Unmanned Aerial Vehicle (UAV) trajectory planning method based on Dijkstra’s algorithm [6]. Despite these improvements, such algorithms remain far from meeting the specific requirements of our scenario. As a result, both A* and Dijkstra are not well-suited for path planning in the joint space of robotic manipulators.

The second category includes sampling-based algorithms, such as Rapidly-exploring Random Tree (RRT) [7] and Probabilistic Roadmap (PRM) [8,9], along with their optimized variants. Compared to the first category, these algorithms are more suitable for high-dimensional spaces and complex environments. This is because sampling-based methods avoid the explicit construction of the configuration space and do not rely on exhaustive enumeration or global discretization, which become computationally prohibitive as the dimensionality increases. Instead, they efficiently explore the space by randomly sampling feasible configurations, which makes them more scalable and adaptable in high-dimensional planning problems. Zhang et al. [10] introduce SVF-RRT* based on RRT*, which speeds up the convergence speed and smoothes the path. Moon et al. [11] proposed a dual-tree RRT to increase the quality of the trajectory. Fan et al. [12] further enhanced RRT* with a goal-biased strategy and a modified artificial potential field, significantly improving convergence rate and path quality in cluttered environments. But most of the improved sampling process introduces a degree of randomness, which will have high time complexity in the high-dimensional space. Novosad et al. Improved PRM to find different paths in complex 3D environment [13]. However, the quality of the generated paths is often suboptimal, as they only provide asymptotically optimal solutions. Overall, while improved versions of RRT and PRM offer better performance, they still face limitations in efficiency and path quality, especially in high-dimensional planning scenarios. Despite these challenges, the inherent scalability and flexibility of sampling-based algorithms make them a promising foundation for further exploration and enhancement in high-dimensional motion-planning problems.

The third category consists of optimization-based methods, including Artificial Potential Field (APF) [14], Particle Swarm Optimization (PSO) [15,16,17], and SA (Simulated Annealing) [18,19]. For example, Wang et al. proposed an APF-CPP approach, in which they designed a unique coverage strategy to efficiently explore the environment [20]. Similarly, Yang et al. introduced an adaptive APF method to generate smoother trajectories in cluttered environments [21]. However, most of these improved methods are designed for 3D task spaces. In high-dimensional joint spaces, APF-based approaches typically lack a sufficient global perspective, making it difficult to maintain path consistency and avoid local extremes effectively. For PSO and SA methods, due to the large number of iterations required to sufficiently explore the configuration space, they are generally not considered for our scenario.

The fourth category comprises deep learning (DL) and reinforcement learning (RL)-based methods [22,23,24,25]. With recent advancements in artificial intelligence, approaches that integrate RL with classical planners such as RRT or PRM have gained increasing attention in path-planning applications. For instance, Francis et al. combined PRM with RL to achieve efficient indoor navigation for mobile robots [26], while Liu et al. integrated RRT with DL to enhance path exploration efficiency [27]. However, these learning-based methods typically require large amounts of training data, and their applicability to real-time computation remains a significant challenge. Furthermore, they often have problems with unstable training results and struggle to incorporate joint constraints or kinematic constraints, which limits their effectiveness in complex robotic manipulator scenarios.

Building upon the comparison of different types of planning algorithms aforementioned, this paper selects sampling-based algorithms for path planning in the joint space of robotic manipulators. In recent years, RRT has been one of the most classical path-planning algorithms and has been continuously improved to enhance its performance and computational efficiency. Among these improvements, the RRT-Connect algorithm [28] is a significant variant that employs a bidirectional expansion strategy, where two trees are grown simultaneously from the start and goal points. The second tree continuously expands toward the first tree until they connect. This approach incorporates greedy search principles, which accelerate convergence and improve search efficiency. Additionally, there are sampling-guided optimization algorithms [29,30] that refine the sampling strategy to guide tree growth more effectively, thereby expediting convergence and reducing ineffective sampling during exploration. However, it is important to note that many RRT-based improvements were not specifically designed for manipulators, and directly applying them to path planning in the joint space of manipulators presents several challenges. For instance, manipulators are constrained by joint angle limitations, and path planning must strictly adhere to these constraints to prevent mechanical structure conflicts or infeasible inverse kinematics (IK) solutions. IK refers to the process of computing the joint angles of a manipulator that will place its end-effector at a desired position and orientation in space. Solving IK is often challenging due to the non-linear relationships between joint variables and end-effector pose, and because multiple or even no valid solutions may exist. Moreover, due to the high degrees of freedom and redundancy of manipulators, path planning in joint space requires extensive computation and long planning times, making it difficult to meet real-time requirements. Therefore, further optimization and refinement of path-planning algorithms are necessary to improve efficiency and accommodate engineering applications.

**Figure 1:**
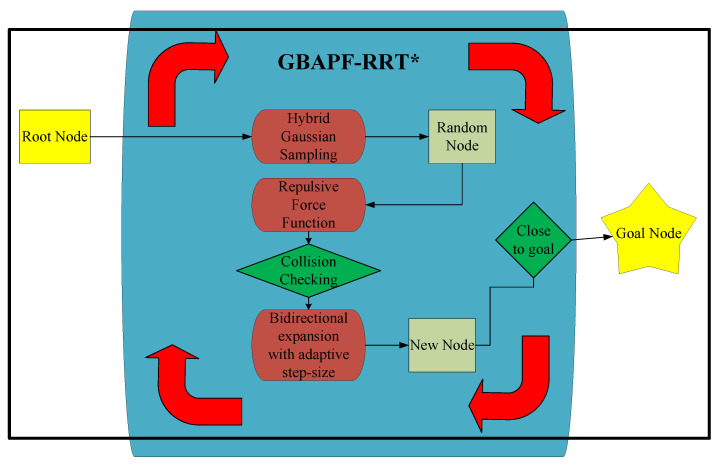
Schematic diagram of system framework.

Based on the aforementioned research findings and the limitations of existing algorithms in joint space path planning for manipulators, this paper aims to address key challenges such as convergence speed, obstacle avoidance, and path smoothness. To this end, we propose an optimized goal-biased bidirectional artificial potential field rapidly-exploring random tree star (GBAPF-RRT*) algorithm that enhances computational efficiency and planning quality in high-dimensional spaces, making the following key contributions and overall framework of the proposed algorithm is illustrated in Figure 1:

1. Hybrid Gaussian Sampling Method—A hybrid Gaussian distribution is employed to optimize the sampling process, effectively guiding tree expansion toward the target point. This approach improves convergence speed, minimizes unnecessary sampling nodes, and enhances search efficiency. Compared to unguided RRT, this method significantly improves the efficiency of tree expansion and increases the likelihood of finding a feasible path within limited iterations.
2. Modified Repulsive Force Function—While the original APF method is not well-suited for path planning in high-dimensional space like joint space, an improved repulsive force function is integrated into the expansion process. This enhancement significantly improves obstacle avoidance performance in high-dimensional joint spaces, making it more effective for robotic manipulator path planning.
3. Bidirectional expansion with adaptive step-size—A bidirectional tree expansion approach is adopted, allowing two trees to grow simultaneously and efficiently connect. Additionally, an adaptive step-size strategy mitigates local extreme issues, ensuring a more stable and flexible search process in complex environments. Compared to conventional single-tree expansion, this bidirectional strategy significantly accelerates the search process and improves pathfinding efficiency in high-dimensional spaces.

## 2. Related Work

Although RRT and its variants are well-established in the path-planning literature, we briefly revisit the basic RRT family of algorithms for two key reasons. First, our proposed method builds directly upon the foundational RRT framework, and understanding its core mechanisms helps clarify how our contributions extend and improve the original formulation. Second, to fairly evaluate the performance of our enhancements, it is essential to compare them against the baseline behaviors of classical algorithms such as RRT and RRT*. Therefore, we provide a concise review of these representative methods to establish the necessary context for our improvements.

### 2.1. Basic Algorithm

#### 2.1.1. RRT

RRT is a classic and widely used algorithm in the field of path planning [7]. It finds a feasible path from the start to the goal by randomly sampling points on the map and expanding the tree accordingly.

The algorithm begins by randomly sampling a point in the space. Then it moves from the nearest existing node in the tree toward the sampled point by a step size. The new node at the new position will connect to the previous node. If the newly created node has not yet reached the goal position, repeat the above steps until a feasible path is found.

However, RRT also has some notable drawbacks. First, since the sampling process is entirely random, the tree may grow in directions that do not necessarily lead toward the goal, resulting in a large number of unnecessary searches and reducing algorithm efficiency. This issue becomes even more prominent in higher-dimensional spaces, such as the joint space of robotic manipulators discussed in this paper. Additionally, in environments with dense or complex obstacles, the randomness of RRT can lead to excessive computational overhead when navigating around obstacles. If the path is surrounded by obstacles, the algorithm may generate many ineffective expansions, significantly reducing the tree’s growth efficiency. In such cases, RRT may require a large number of additional iterations to find a feasible path. Moreover, the generated path is often not optimal and typically requires further post-processing and optimization.

#### 2.1.2. RRT Star

**Figure 2:**
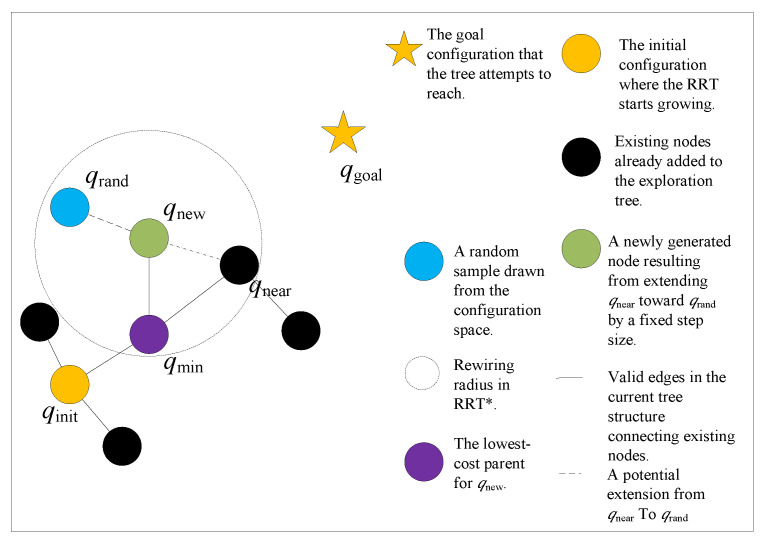
Schematic diagram of RRT*.

RRT* is an enhanced version of the standard RRT algorithm [31]. While the traditional RRT connects each newly generated node directly to its nearest neighbor in the tree, RRT* introduces an optimization step that significantly improves the quality of the resulting path. Instead of blindly attaching the new node to the closest existing node, RRT* searches within a defined neighborhood around the new node to evaluate potential parent nodes. It then selects the parent that results in the lowest cost path to the root in terms. This re-wiring process allows RRT* to refine the path over time. As a result, RRT* not only ensures path feasibility but also enhances path optimality compared to standard RRT. The specific mechanism of RRT* is illustrated in Figure 2.

However, RRT* still does not provide any guidance for selecting the random nodes, meaning the sampling efficiency would decrease significantly in complicated or high-dimensional maps. However, RRT* significantly improves path quality compared to standard RRT by optimizing path connections and rewiring strategies. The resulting paths are smoother and have lower costs, making RRT* more feasible and applicable in practical scenarios.

### 2.2. Improved Version

#### 2.2.1. Dual-Tree Methods

Bidirectional RRT is an extension of the standard RRT algorithm that improves search efficiency by simultaneously growing two trees: one rooted at the start configuration and the other at the goal configuration. These two trees explore the space independently and attempt to connect with each other during the planning process. This strategy reduces search time and increases the likelihood of finding a feasible path in complex or high-dimensional environments. And RRT-Connect is a well-known and more aggressive implementation of the bidirectional RRT strategy [28]. Unlike basic bidirectional RRT, which typically extends trees by small steps, RRT-Connect employs a greedy approach that attempts to fully extend one tree toward the other in a single iteration until it reaches an obstacle or connects. This results in faster convergence and fewer required samples, making RRT-Connect particularly effective in environments with relatively simple obstacle distributions.

#### 2.2.2. Goal-Biased Methods

The goal-biased method introduces an effective improvement to the original RRT framework by incorporating probabilistic guidance toward the goal configuration during sampling [10,14]. Specifically, during each iteration, a certain probability is assigned to directly sample the goal or a point near it. This significantly reduces the number of iterations required to reach a feasible solution, especially in environments with high dimensions.

#### 2.2.3. Modified APF Methods

Although the basic principles of APF have been widely discussed, including in our introductory review, we briefly revisit APF here due to its relevance to our proposed method. Traditional APF approaches are known for their simplicity and real-time responsiveness, but they often encounter difficulties such as local extreme and poor global guidance in high-dimensional spaces.

Recent studies have proposed several improvements to overcome these issues, such as integrating APF with sampling-based methods, dynamic repulsive fields, or adaptive potential functions. These strategies aim to retain the efficiency of APF while improving its global planning capability [20,21]. Inspired by this line of work, our algorithm incorporates a modified APF module to guide local sampling and enhance obstacle avoidance.

## 3. Path-Planning Algorithm Design

### 3.1. Problem Formulation in Joint Space

Firstly, this paper defines the path-planning problem in the joint space. In joint space, each degree of freedom of the robotic manipulator, including all movable joints and the end-effector, is represented as one dimension. As defined in Equation (1), each node *q* is a set of joint angles with a dimension of *d*, where $q\in R^{d}$ is treated as a column vector after transposition, and *d* represents the number of DOF of the manipulator.

Each node *q* in the tree structure corresponds to a specific configuration of the manipulator in the joint space. The goal point $q_{\mathrm{final}}$ is defined as a set of joint angles, representing the desired final pose of the manipulator. The root node $q_{\mathrm{root}}$ of the tree is set to the initial configuration.

By continuously expanding the tree and searching for a feasible path, the algorithm ultimately outputs a sequence of nodes, where each node contains a set of joint angles. This sequence clearly describes the manipulator’s trajectory from the starting configuration to the target configuration and provides a well-defined input for subsequent motion control.

(1) $$q={\left[\begin{array}{cccc} q^{1} & q^{2} & \ldots & q^{d} \end{array}\right]}^{T}$$

### 3.2. Hybrid Gaussian Sampling Method

In the sampling process, this paper introduces a Gaussian distribution-based guided sampling strategy on top of the traditional RRT framework. The Gaussian distribution is a common and practical continuous probability distribution, and its probability density function is defined in (2):

(2) $$f\left(x\right)=\frac{1}{\sigma \sqrt{2\pi }}\exp\left(-\frac{{\left(x-\mu \right)}^{2}}{2\sigma^{2}}\right).$$

where μ determines the center position of the distribution. The variance $\sigma^{2}$ defines the spread and dispersion range of the distribution. A larger variance results in a wider sampling range, providing stronger global exploration ability for the algorithm; conversely, a smaller variance causes the samples to be more concentrated around the mean, favoring local search and fine-tuning.

In this study, the mean μ is set to the target point $q_{g}$, and each joint angle is sampled around this center. In the joint space, the joint angles corresponding to each node will gradually approach the target configuration in a collision-free manner. The value of σ is dynamically adjusted according to the specific situation encountered during the path-planning process. When the algorithm encounters more complex or obstacle-dense environments, σ will be increased accordingly, allowing the sampling points to cover a wider area and enhancing the exploration capability. This helps to prevent the algorithm from becoming trapped in local regions.

Compared to traditional uniform random sampling, the Gaussian-guided sampling method proposed in this paper significantly improves the convergence speed of the algorithm in high-dimensional joint space. It increases the probability of effective expansions toward the target region while reducing invalid searches and collision failures, thereby enhancing the overall efficiency of the path-planning process.

### 3.3. Modified Repulsive Force Function

Although the aforementioned Gaussian-guided sampling method can improve the directional tendency of sampling points and make them more concentrated around the target, it does not inherently introduce explicit obstacle avoidance capability. In complex environments or high-dimensional joint spaces, relying solely on random or Gaussian sampling still tends to produce a large number of invalid sampling points that fall into obstacle regions, leading to low search efficiency. The algorithm may get stuck around obstacles and fail to quickly find a feasible path.

To address this issue, this paper further introduces the repulsive force function from the APF method into the expansion strategy and modifies it to be applicable in high-dimensional joint spaces. The mechanism of the repulsive force is to generate a “repelling effect” around obstacle regions, causing sampling points to be generated further away from obstacles. This effectively reduces the probability of sampling points falling into obstacle regions or near obstacle boundaries.

In traditional APF methods, the repulsive force function typically increases in an exponential or quadratic form concerning the distance between the sampling point and the obstacle. However, directly applying such functions in high-dimensional spaces can lead to excessive computational overhead or cause large gradient oscillations. In this work, based on the characteristics of the joint space, the repulsive force function is carefully adjusted in terms of parameters and functional form. This ensures that it retains effective obstacle avoidance capability while avoiding overly strong repulsive forces that could lead to unstable or oscillatory paths. In this way, the sampling points can maintain goal-directed guidance while also acquiring a certain level of obstacle avoidance ability, thereby further improving the search efficiency and robustness of the algorithm in complex, high-dimensional environments.

The first point to note is that in joint space, variations in joint angles do not have a linear correspondence with the end-effector’s position in Cartesian space. In other words, increasing or decreasing a joint angle does not directly result in the end-effector moving away from or closer to an obstacle. Therefore, traditional repulsive force functions based on Euclidean distance cannot be directly applied in joint space.

In joint space, simply adjusting a single joint angle does not achieve the same “pushing away” effect as in Cartesian space, and may even lead to movement directions contrary to the desired obstacle avoidance behavior. Therefore, it is necessary to redesign and adjust the form and action mechanism of the repulsive force function to make it suitable for path planning in high-dimensional joint space.

Accordingly, this paper initially defines the repulsive force function for each joint in joint space as Equation  (3):

(3) $$U_{\mathrm{rep},i}=\left\{\begin{array}{cc} \frac{1}{2}k_{\mathrm{rep}}\cdot {\left(\frac{1}{d_{i}\left(q\right)}-\frac{1}{d_{0}}\right)}^{2}, & d_{i}\left(q\right)\leq d_{0}\\ 0, & d_{i}\left(q\right)>d_{0} \end{array}\right.$$

where $k_{\mathrm{rep}}$ is the repulsive force coefficient, which can be adjusted according to specific application scenarios and obstacle avoidance requirements. $d_{i}\left(q\right)$ represents the distance from the *i*-th point of the current joint configuration to the nearest obstacle, and $d_{0}$ denotes the effective range of the repulsive force. When the distance is less than or equal to $d_{0}$, the repulsive force function takes effect; when the distance exceeds $d_{0}$, the repulsive force becomes zero. A smaller repulsive range $d_{0}$ helps to prevent the same joint from being influenced by conflicting repulsive forces from multiple obstacles simultaneously, thus avoiding confusion in force direction and ensuring the clarity and stability of the repulsive effect. This contributes to a smoother and more reliable obstacle avoidance process in the joint space.

In addition, it is important to address the directionality issue mentioned earlier. In joint space, simply increasing or decreasing joint angles does not allow one to directly determine whether such adjustments will move the end-effector or joint position away from obstacles. Since there is no linear relationship between joint angle changes and the corresponding position in Cartesian space, an incorrect adjustment direction may cause the sampling point to move closer to the obstacle, increasing the risk of collision. To address this, a disturbance-based direction verification mechanism is introduced in this paper. Before each sampling or joint angle adjustment, the Cartesian position of the joint must first be calculated, as shown in Equation (4). By obtaining the joint’s position in Cartesian space and evaluating distance changes under small perturbations, the algorithm can determine whether the current adjustment direction is beneficial for moving away from the obstacle, thereby ensuring the correctness and effectiveness of the obstacle avoidance direction.

(4) $$p_{i}=\left[\begin{array}{c} x_{i}\\ y_{i}\\ z_{i} \end{array}\right]=\left[\begin{array}{cc} I_{3} & 0 \end{array}\right]\cdot \left(\prod_{k=1}^{i}T_{k-1}^{k}\left(q_{k}\right)\right)\cdot \left[\begin{array}{c} 0\\ 0\\ 0\\ 1 \end{array}\right]$$

where $p_{i}\in R^{3}$ denotes the position of the *i*-th joint in the world coordinate frame, and $\left(x_{i},y_{i},z_{i}\right)$ are its Cartesian coordinates. Each $T_{k-1}^{k}\left(q_{k}\right)\in SE\left(3\right)$ represents the homogeneous transformation matrix from frame k−1 to frame *k*, determined by the joint variable $q_{k}$. The cumulative transformation from the base to the *i*-th joint is obtained through the ordered product $\prod_{k=1}^{i}T_{k-1}^{k}\left(q_{k}\right)$. The resulting transformation is applied to the local origin point ${\left[0\;0\;0\;1\right]}^{T}$, and the final position $p_{i}$ is extracted by projecting the resulting homogeneous vector using the projection matrix $\left[I_{3}\;0\right]$, where $I_{3}$ denotes the 3×3 identity matrix.

Based on this, we further perform disturbance testing on each joint angle to determine the correct adjustment direction. Specifically, a small positive and negative perturbation is applied to each joint, and the resulting change in distance to the nearest obstacle is compared. If the distance to the obstacle increases after a positive perturbation, this direction is considered beneficial for obstacle avoidance, and the sign is set to positive; otherwise, it is set to negative. Finally, the directional signs corresponding to each joint are combined into a direction vector, which is used to guide obstacle avoidance adjustments in the joint space. The definitions are given in Equations (5) and (6):

(5) $${\mathrm{sign}}_{i}=\left\{\begin{array}{cc} +1, & \mathrm{if}\;d\left(q_{i}+\delta \right)>d\left(q_{i}-\delta \right)\\ -1, & \mathrm{otherwise} \end{array}\right.$$

(6) $$\mathrm{dir}={\left[\begin{array}{cccc} {\mathrm{sign}}_{1} & {\mathrm{sign}}_{2} & \ldots & {\mathrm{sign}}_{d} \end{array}\right]}^{T}$$

Therefore, the final repulsive force function is defined in Equation (7):

(7) $$F_{\mathrm{rep},i}=\left\{\begin{array}{cc} k_{\mathrm{rep}}\cdot \left(\frac{1}{d_{i}\left(q\right)}-\frac{1}{d_{0}}\right)\cdot \frac{1}{d_{i}{\left(q\right)}^{2}}\cdot {\mathrm{dir}}_{i}, & d_{i}\left(q\right)\leq d_{0}\\ 0, & d_{i}\left(q\right)>d_{0} \end{array}\right.$$

The second issue that needs to be addressed arises from the structure of the robotic manipulator. In most cases, changing the angle of the current joint $q_{i}$ does not directly alter the Cartesian position of that joint itself. To achieve positional changes of the current joint in Cartesian space, it is necessary to adjust the angle of the preceding joint. Therefore, the repulsive force ultimately acts on the (i−1)-th joint. The corresponding repulsive force is defined in Equation (8):

(8) $$F_{\mathrm{rep},\;\mathrm{link},i-1}+=k_{\mathrm{rep}}\cdot \left(\frac{1}{d_{\mathrm{link}}}-\frac{1}{d_{0}}\right)\cdot \frac{1}{d_{\mathrm{link}}^{2}}\cdot {\mathrm{dir}}_{\mathrm{link}}$$

where $d_{\mathrm{link}}$ denotes the minimum distance between the discretized sampling point on the link and the obstacle, and ${\mathrm{dir}}_{\mathrm{link}}$ is the optimal avoidance direction calculated through bidirectional perturbation testing.

In addition, there are more stringent constraints to be considered. For certain manipulator structures, adjusting only the previous joint angle generally allows changes in the vertical (up-and-down) direction. To generate an effective lateral repulsive force, the angle of the base joint would need to be adjusted. However, this paper does not adopt such a method, since applying excessive repulsive forces onto a single joint often leads to instability and prevents achieving the desired obstacle avoidance effect.

**Figure 3:**
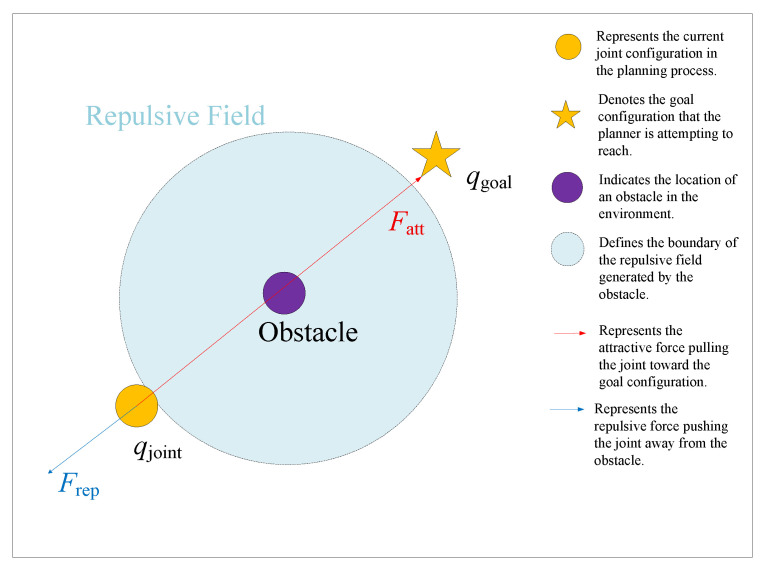
An illustration of the local extreme problem in the APF method.

The third issue is the local extreme problem, which is a common challenge in the APF method, as shown in Figure 3. When the robot, the obstacle, and the goal point lie on the same straight line, the attractive force and the repulsive force may reach a balance at a certain point, resulting in a net force of zero. However, at this equilibrium point, the sampling node remains near the goal region without progressing toward the target. In this situation, the robot is unable to continue moving toward the goal, causing the algorithm to become stuck and unable to escape from the local extreme, thus negatively impacting the feasibility and global optimality of the path-planning process.

**Figure 4:**
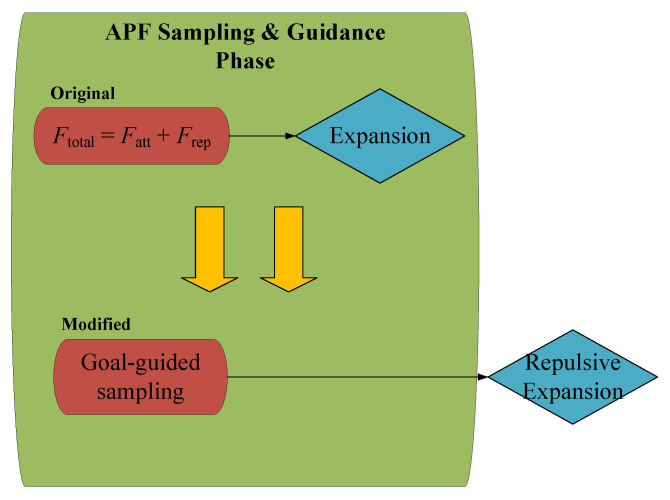
An illustration of the APF Sampling and Guidance Phase break into two parts.

To address this issue, in this paper, the repulsive force computation is incorporated into the expansion step rather than the sampling step. Specifically, the algorithm first performs Gaussian-distributed random sampling around the goal point and then applies the repulsive force adjustment during the node expansion phase. This modifies the original APF formulation:

(9) $$F_{\mathrm{total}}=F_{\mathrm{att}}+F_{\mathrm{rep}}$$

and transforms it into a two-step process: one part is guided by the goal point by the proposed goal-biased method, and the other part is a local adjustment to avoid obstacles based on repulsive force feedback, as illustrated in Figure 4. By adopting this approach, each expansion step is influenced by both the sampling point and the distance to the goal, dynamically adjusting the direction of tree growth. Because the process of adding the attractive and repulsive forces has been canceled, this significantly reduces the occurrence of local extreme and improves the robustness of the algorithm.

### 3.4. Expansion Strategy

To improve the search efficiency, the proposed algorithm adopts a bidirectional tree expansion strategy (Bidirectional RRT) for path construction. Tree A is rooted at the start configuration in the joint space and extends towards the direction of the goal configuration. Conversely, Tree B is rooted at the goal configuration and expands in the direction guided by the latest node of Tree A. Unlike the traditional RRT-Connect method, the proposed algorithm expands each tree only once in every iteration. This strategy effectively prevents Tree B from getting trapped in local regions with repeated iterations in complex environments, thereby enhancing the overall success rate and efficiency of path planning.

Compared to the traditional unidirectional RRT, the dual-tree strategy does not incur additional computational complexity in theory. Both methods have an approximate time complexity of O(NlogN), where *N* is the number of sampled nodes and logN accounts for nearest-neighbor search. In practice, however, the proposed bidirectional approach often exhibits superior computational efficiency. This is primarily because two trees collaboratively explore the configuration space and tend to connect more rapidly, especially in high-dimensional or cluttered environments. Furthermore, by limiting each tree to a single expansion per iteration, the proposed strategy reduces redundant explorations and avoids unnecessary growth in confined regions, thus accelerating convergence and improving the success rate without introducing a performance loss.

**Figure 5:**
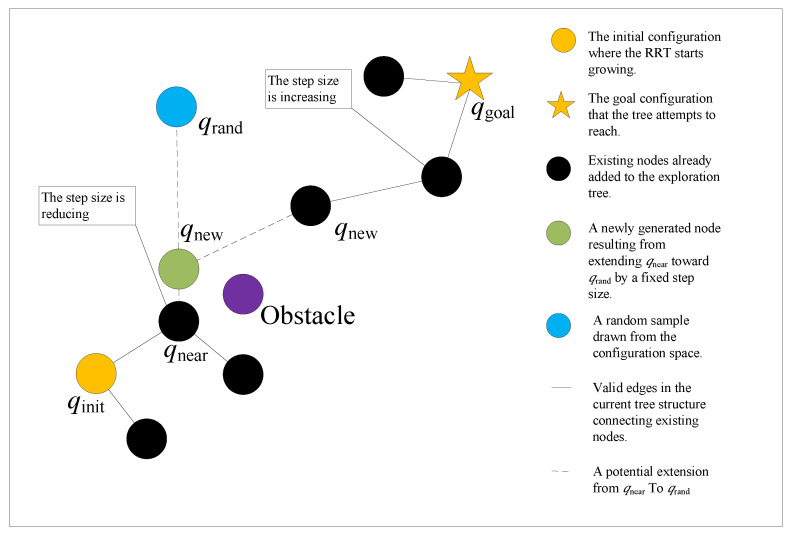
Schematic of the proposed bidirectional expansion strategy.Tree A starts from the initial configuration and grows toward the goal, while Tree B grows from the goal towards the latest node of Tree A. Each tree expands only once per iteration to avoid repeated expansion failures in local regions. An adaptive step-size mechanism is also employed to accelerate expansion in open space and improve precision near obstacles.

To further enhance the performance of bidirectional expansion, an adaptive step-size strategy is integrated into the planning process. The step size δ is initially set to 0.2, allowing for faster expansion in open regions when no collisions are detected. As the collision counter increases, δ is gradually reduced to 0.1 to enable more precise motion near obstacles, thereby improving local adaptability and collision avoidance, as illustrated in Figure 5.

### 3.5. Collision Checking Strategy

In our implementation, collision checking is based on evaluating joint configurations via forward kinematics to obtain the corresponding Cartesian positions of the manipulator’s joints and links. Since obstacles are defined in a 3D workspace, collision evaluation is ultimately conducted in Cartesian space. For each sampled configuration, the 3D positions of all six joints are computed, and two levels of collision checking are applied. First, each joint position must maintain a minimum clearance from all obstacles. Second, each link is modeled as the straight segment between two adjacent joints and checked to ensure that all points along the link maintain a specified minimum distance from obstacles. During the RRT extension process, dense interpolation in joint space is performed, and each intermediate configuration is validated through forward kinematics for both joint and link collision clearance. These clearance thresholds can be tuned according to the actual dimensions of the manipulator’s joints and links. Only when all intermediate steps satisfy the clearance constraints will the extension be accepted. This ensures that the entire planned trajectory remains collision-free with respect to the manipulator’s real physical model.

The final form of the proposed GBAPF-RRT* algorithm is described in Algorithm 1.
**Algorithm 1** The proposed GBAPF-RRT***Require:** Start configuration $q_{\mathrm{start}}$, goal configuration $q_{\mathrm{goal}}$, maximum iterations *N*, step size Δq, threshold ε

**Ensure:** A feasible path from $q_{\mathrm{start}}$ to $q_{\mathrm{goal}}$ (if found)
1:Initialize trees: $T_{A}\leftarrow \left\{q_{\mathrm{start}}\right\}$, $T_{B}\leftarrow \left\{q_{\mathrm{goal}}\right\}$2:**for** i=1 to *N* **do**                       ▹ — Expand Tree A —3:      $q_{\mathrm{rand}}^{A}\leftarrow \mathrm{GaussianSample}\left(q_{\mathrm{goal}},\sigma \right)$4:      $q_{\mathrm{near}}^{A}\leftarrow \mathrm{Nearest}\left(T_{A},q_{\mathrm{rand}}^{A}\right)$5:      $q_{\mathrm{new}}^{A}\leftarrow \mathrm{extend}\left(q_{\mathrm{near}}^{A},q_{\mathrm{rand}}^{A},\Delta q\right)$6:      **if not** $\mathrm{CollisionFree}(q_{\mathrm{near}}^{A},q_{\mathrm{new}}^{A})$ **then**7:            **continue**8:       **end if**9:       $q_{\mathrm{new}}^{A}\leftarrow q_{\mathrm{new}}^{A}+F_{\mathrm{rep}}\left(q_{\mathrm{new}}^{A}\right)$                ▹ Apply repulsive force10:      Add $q_{\mathrm{new}}^{A}$ to $T_{A}$, connect with $q_{\mathrm{near}}^{A}$                            ▹ — Expand Tree B —11:       $q_{\mathrm{rand}}^{B}\leftarrow \mathrm{GaussianSample}\left(q_{\mathrm{new}}^{A},\sigma \right)$12:       $q_{\mathrm{near}}^{B}\leftarrow \mathrm{Nearest}\left(T_{B},q_{\mathrm{rand}}^{B}\right)$13:       $q_{\mathrm{new}}^{B}\leftarrow \mathrm{extend}\left(q_{\mathrm{near}}^{B},q_{\mathrm{rand}}^{B},\Delta q\right)$14:       **if not** $\mathrm{CollisionFree}(q_{\mathrm{near}}^{B},q_{\mathrm{new}}^{B})$ **then**15:            **continue**16:       **end if**17:       $q_{\mathrm{new}}^{B}\leftarrow q_{\mathrm{new}}^{B}+F_{\mathrm{rep}}\left(q_{\mathrm{new}}^{B}\right)$18:       Add $q_{\mathrm{new}}^{B}$ to $T_{B}$, connect with $q_{\mathrm{near}}^{B}$                             ▹ — Try to Connect —19:       **if** $\mathrm{Distance}(q_{\mathrm{new}}^{A},q_{\mathrm{new}}^{B})<\varepsilon$ **then**20:            **return** $\mathrm{ExtractPath}(T_{A},T_{B})$21:       **end if**22:**end for**23:**return** Failure


## 4. Probabilistic Completeness

Probabilistic completeness is defined as the property that, if a feasible collision-free path exists in the current environment, the algorithm is guaranteed to find such a path given infinite iterations.

In the proposed algorithm, a Gaussian-based sampling strategy is adopted, where samples are drawn around the goal configuration $q_{G}$ according to (2).

According to this formulation, as the standard deviation σ increases, the distribution becomes flatter and the sampling range expands. Theoretically, when σ→∞, the Gaussian distribution approximates a uniform distribution within the joint space limits.

Under this condition, the sampling mechanism effectively degenerates into uniform exploration over the free configuration space, thus aligning with the classical probabilistic completeness guarantees of RRT and RRT*. In our implementation, an adaptive sampling mechanism is introduced: when consecutive sampling attempts fail due to collisions, the value of σ is gradually increased. This enables a smooth transition from goal-biased local exploration to global uniform sampling.

In addition to the Gaussian-based sampling strategy, the proposed GBAPF-RRT* algorithm incorporates a modified repulsive force mechanism inspired by the APF method. It is important to note that the repulsive force adjustment is applied during the expansion phase rather than the sampling phase. Specifically, random sampling remains unbiased and probabilistically complete, ensuring that any region of the free configuration space has a nonzero probability of being sampled over infinite iterations.

The APF-based repulsive correction merely influences the direction of local expansion around sampled points, providing a bias toward obstacle avoidance. However, it does not restrict or exclude any feasible regions from being eventually sampled or explored. Consequently, the global exploratory capability of the algorithm is preserved.

Furthermore, an adaptive mechanism is introduced whereby the influence of the repulsive force diminishes as the sampling radius expands. As the standard deviation σ of the Gaussian distribution increases, the sampling behavior asymptotically approximates uniform random exploration, and the relative effect of the repulsive correction becomes negligible.

Therefore, considering the independence of the sampling process and the non-exclusionary nature of the local expansion adjustment, the proposed GBAPF-RRT* algorithm maintains probabilistic completeness. Given sufficient iterations, it is guaranteed to find a feasible collision-free path with probability one, satisfying the formal definition of probabilistic completeness.

## 5. Simulation

To present the simulation experiments more clearly and systematically, we first describe the simulation setup in Section 5.1. The proposed algorithm is then applied to two distinct scenarios, i.e., complex obstacle cluster.

### 5.1. Simulation Setup

In this study, the base of the manipulator is placed in the center of an obstacle cluster on the map, with the objective of evaluating the algorithm’s performance in obstacle avoidance and path planning from an initial pose to a specified target pose. Two different simulation environments are designed, both within a 50 cm × 50 cm × 50 cm grid space. The first environment features a complex cluster of small obstacles, while the second includes a narrow entrance, simulating challenging terrain and confined spaces commonly encountered in real-world scenarios. All experiments are conducted on a Windows 10 operating system, running on a hardware platform equipped with a 12th Gen Intel Core i7-12700KF processor (base frequency 3.6 GHz) (Santa Clara, CA, USA) and 32 GB dual-channel DDR5 memory.

**Figure 6:**
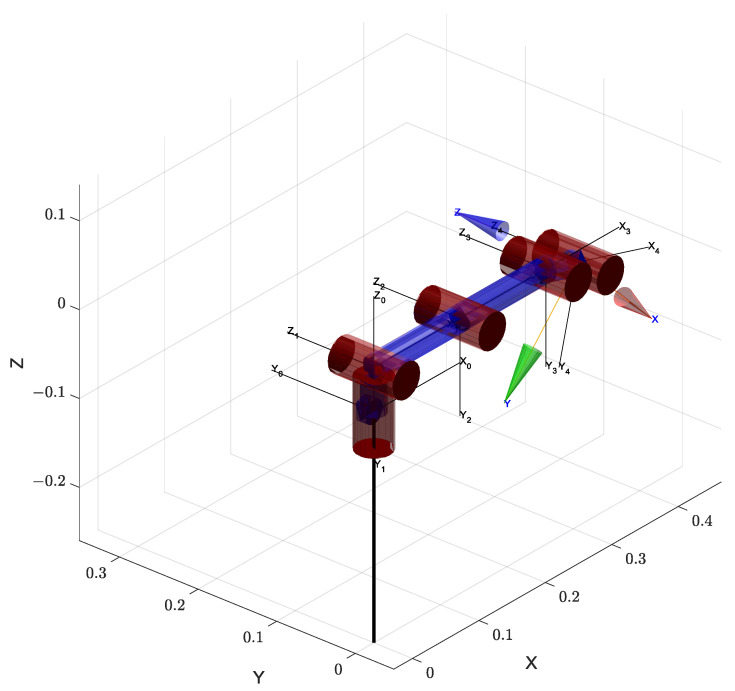
Visualization of the manipulator’s coordinate frames. The coordinate frames shown here are generated by the MATLAB R2024a SerialLink function, which uses a visualization style resembling the modified DH convention. Each joint frame is labeled with its respective $X_{i}$, $Y_{i}$, and $Z_{i}$ axes. Joints 5 and 6 represent the end-effector’s rotational and gripper actuation components, respectively. To improve the clarity of the visualization, the joint angles were adjusted to separate the overlapping frames.

The standard Denavit–Hartenberg (DH) parameters of the manipulator are defined in Table 1, and the joint coordinate systems are illustrated in Figure 6.

In the simulation, we compare four different path-planning algorithms: the standard RRT*, bi-directional RRT*, Goal-Biased RRT*, and the proposed GBAPF-RRT* algorithm. Each algorithm is tasked with planning a collision-free path from a given initial pose to a specified target pose through this complex environment.

Key performance metrics, including planning time, success rate, path length, and the number of sampling nodes are recorded and analyzed. These indicators are used to comprehensively evaluate the performance and applicability of each algorithm under complex obstacle conditions.

Given the high-dimensional, multi-degree-of-freedom, and non-linear constrained nature of the joint space, the complexity of the search process increases significantly. To ensure a fair comparison under consistent conditions, the maximum number of iterations for all planning algorithms is uniformly set to 30,000. If an algorithm fails to find a valid path within this limit, it is deemed a failure case, and the corresponding path length is recorded as zero. This evaluation criterion ensures consistency in statistical analysis and enables a quantitative assessment of failed attempts. In addition, to ensure fairness for different algorithms, all the algorithms adopt the same collision detection function and are tested with identical start and goal configurations. This setup guarantees that the variations of the final result depend only on the performance of the algorithms themselves.

### 5.2. Simulation 1: Complex Obstacle Cluster Environment

**Figure 7:**
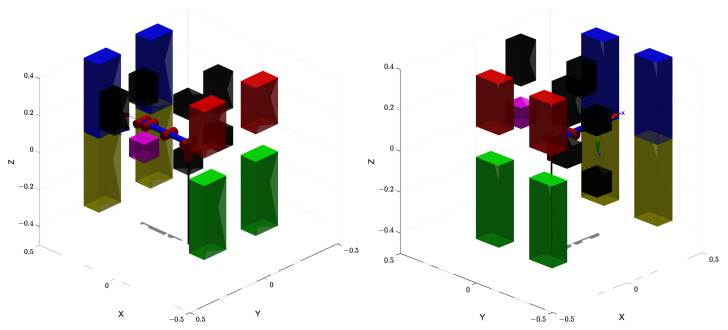
The first simulation map with the manipulator at the start configuration. And the surrounding colored blocks represent static obstacles.

**Figure 8:**
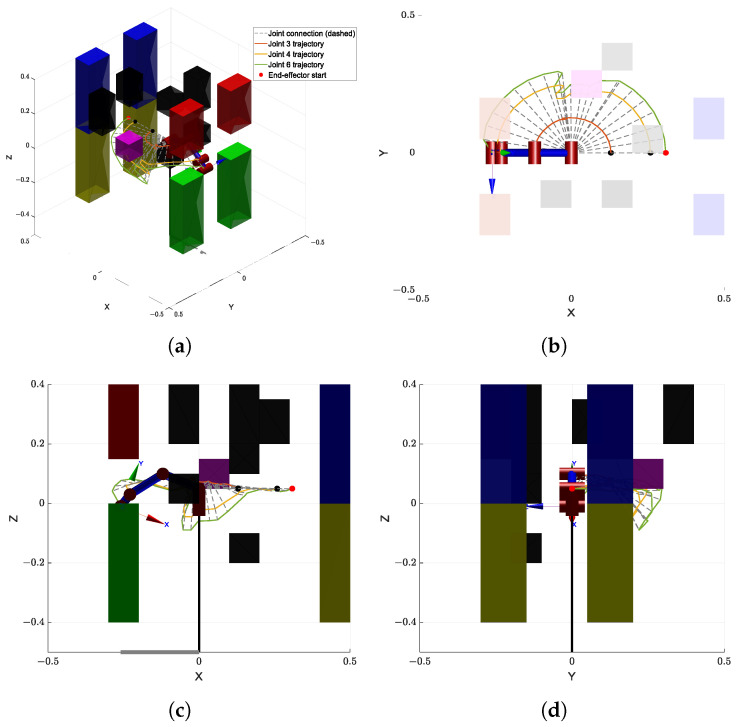
The output trajectories generated by the proposed GBAPF-RRT* algorithm are illustrated, based on the environment setup of Simulation 1. The manipulator is shown at its final goal configuration. Each node of the RRT tree is connected by gray dashed lines to represent the manipulator’s link configurations along the planned path. The orange, yellow, and green solid lines correspond to the trajectories of joint 3, joint 4, and joint 6 (end-effector), respectively. A red dot marks the initial position of the end-effector. The subfigure (**a**) provides a general 3D perspective, while subfigures (**b**–**d**) show the same trajectory projected onto the X–Y, X–Z, and Y–Z planes, respectively.

The first simulation is conducted in an environment composed of a combination of multiple small-scale obstacles and one or more large obstacles, as illustrated in Figure 7. This setup is designed to emulate realistic, cluttered environments that include both densely packed local obstacles and large-scale barriers, thus imposing significant challenges on both global and local path-planning capabilities. Moreover, the visualization of the planned trajectories is illustrated in Figure 8, which clearly shows that the proposed algorithm can effectively achieve obstacle avoidance.

**Figure 9:**
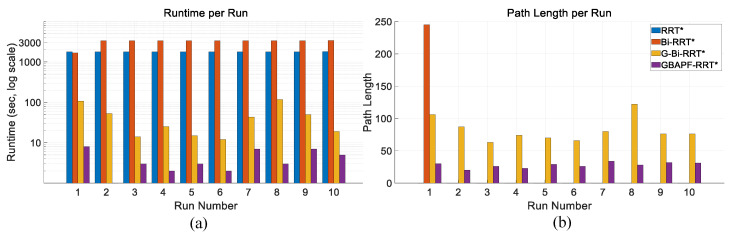
Comparison of runtime (**a**) and path length (**b**) for four algorithms over ten trials. A path length of zero indicates failure to find a valid trajectory. The results are obtained from trials conducted in Simulation 1.

Figure 9a shows the comparison of runtime performance across different algorithms in this simulation environment. The proposed GBAPF-RRT* algorithm clearly outperforms the baseline methods in terms of computational efficiency, demonstrating faster convergence and significantly reduced computation time, particularly in complex obstacle scenarios. These results highlight the effectiveness of the guided sampling strategy and the enhanced node expansion mechanism.

Figure 9b presents the path length comparison among the evaluated algorithms. Although some baseline methods are capable of generating valid paths, they often produce unnecessary detours or redundant segments, leading to longer path lengths. In contrast, the GBAPF-RRT* algorithm consistently produces shorter and smoother trajectories while maintaining a high success rate. This demonstrates its superior search quality and implicit path optimization ability. Furthermore, smooth and concise paths are particularly beneficial for practical manipulator motion execution, as they enhance motion continuity, reduce energy consumption, and ultimately improve the overall applicability of the proposed algorithm.

### 5.3. Simulation 2: Constrained Manipulation Through Structural Openings

**Figure 10:**
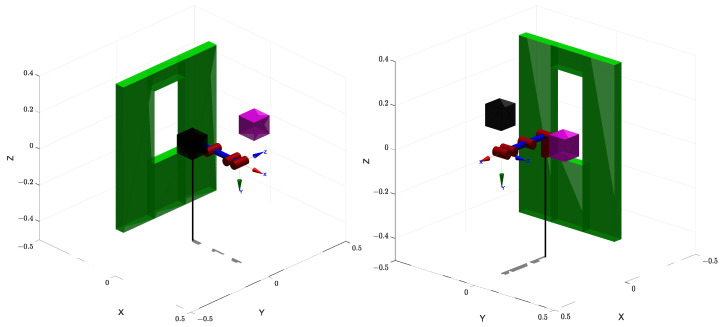
The second simulation map with the manipulator at the start configuration. And the surrounding colored blocks represent static obstacles.

**Figure 11:**
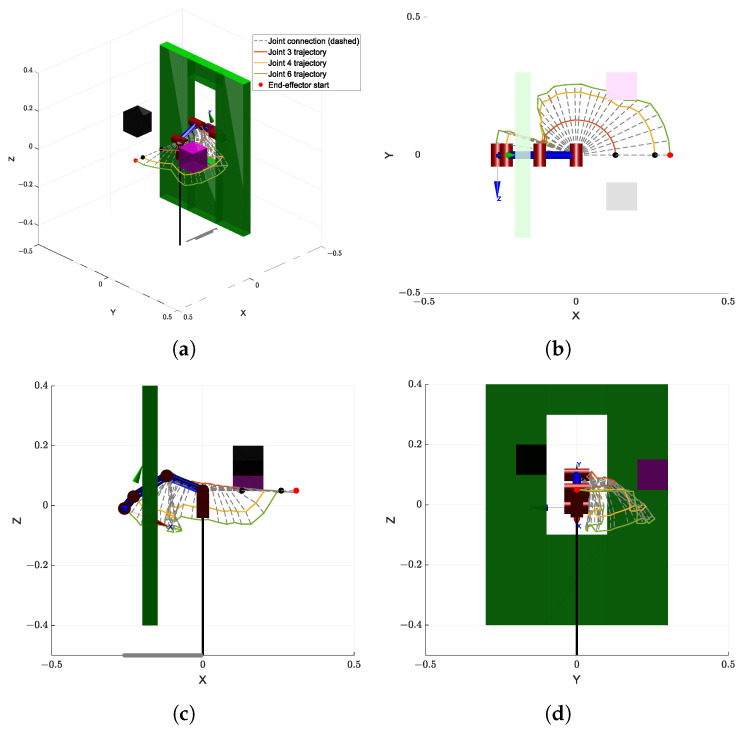
The output trajectories generated by the proposed GBAPF-RRT* algorithm are illustrated, based on the environment setup of Simulation 2. The manipulator is shown at its final goal configuration. Each node of the RRT tree is connected by gray dashed lines to represent the manipulator’s link configurations along the planned path. The orange, yellow, and green solid lines correspond to the trajectories of joint 3, joint 4, and joint 6 (end-effector), respectively. A red dot marks the initial position of the end-effector. The subfigure (**a**) provides a general 3D perspective, while subfigures (**b**–**d**) show the same trajectory projected onto the X–Y, X–Z, and Y–Z planes, respectively.

The second simulation scenario is designed in an environment featuring a vertical structural obstacle with a rectangular opening, as shown in Figure 10. The corresponding planned trajectories are visualized in Figure 11. This setup is intended to simulate realistic operational conditions where a manipulator must reach through constrained passages or apertures in structural elements. Such environments are commonly encountered in industrial tasks involving access through maintenance windows, workstation cutouts, or openings in protective housings for tasks such as assembly, inspection, or pick-and-place operations.

Compared to open-space navigation, this type of task introduces additional challenges, such as restricted entry space, higher motion precision requirements, and tighter constraints on the end-effector’s orientation. Successfully navigating through narrow openings necessitates enhanced robustness in path planning and greater spatial accuracy, particularly in confined, cluttered, or semi-enclosed environments.

**Figure 12:**
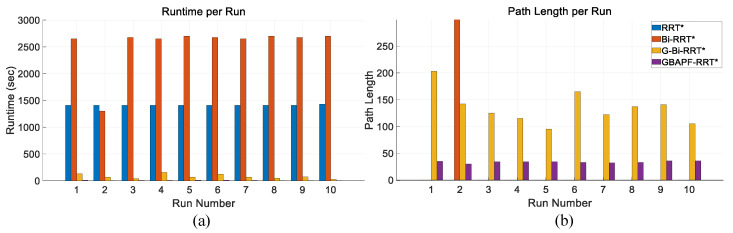
Comparison of runtime (**a**) and path length (**b**) for four algorithms over ten trials. A path length of zero indicates failure to find a valid trajectory. he results are obtained from trials conducted in Simulation 2.

Figure 12a presents a comparison of the computation times of each algorithm in this simulation environment. It can be clearly seen that the proposed GBAPF-RRT* algorithm maintains a significant advantage in time efficiency, generating feasible paths within shorter durations. In scenarios with narrow openings and limited maneuvering space, its guided sampling mechanism effectively enhances search directionality and accelerates convergence.

Additionally, Figure 12b shows the path length comparison across the algorithms. While some traditional methods are able to complete the task, the resulting paths often include unnecessary detours or lack smoothness, compromising overall path quality. In contrast, GBAPF-RRT* consistently achieves high success rates while generating more compact and continuous trajectories. This contributes to lower control complexity and energy consumption during execution, further highlighting the algorithm’s strength in path optimization and its practical value in constrained robotic applications.

### 5.4. Results and Discussion

Table 2 presents the experimental results for the Simulation 1 map. In terms of runtime, it can be observed that as the algorithms evolve progressively based on the original RRT* framework, their performance improves accordingly. To reflect the real-time requirements in practical applications, all algorithms were constrained with a maximum of 30,000 iterations. Consequently, some algorithms failed to generate a valid collision-free path within this limit in certain runs. With respect to path length, the proposed algorithm demonstrates a significantly shorter average path, reducing path length by approximately 60% compared to conventional goal-biased bidirectional algorithms, while maintaining a high success rate.

Table 3 shows the results for the Simulation 2 map. In this more challenging scenario, the runtime of all algorithms increased substantially. This is mainly due to the higher number of required samples when navigating through narrow passages in joint space to ensure collision-free paths. Moreover, the parent rewiring mechanism in RRT* under dense sampling conditions contributes to a considerable increase in computational cost. Despite these challenges, the proposed GBAPF-RRT* algorithm still outperforms the other methods in both path length and success rate, demonstrating superior robustness and adaptability in constrained environments.

**Figure 13:**
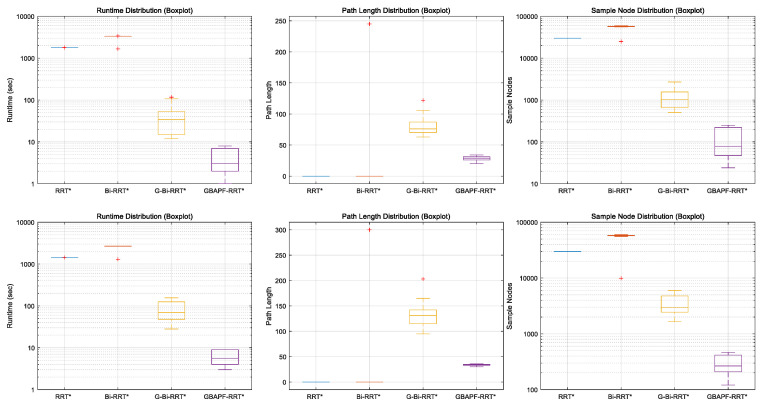
Boxplots of runtime, path length, and sample nodes for all algorithms. The top row corresponds to Simulation 1 and the bottom row to Simulation 2. Red “+” symbols indicate outliers.

To further analyze the stability and variance of different algorithms, boxplots of runtime, path length, and sample nodes are presented in Figure 13. The top row corresponds to Simulation 1, while the bottom row shows the results from Simulation 2. From the plots, it is evident that GBAPF-RRT* not only achieves the lowest median runtime but also exhibits the smallest interquartile range in both maps, indicating high stability and consistency. In contrast, G-Bi-RRT* displays a larger variance in runtime and sample nodes, especially in the more complex Simulation 2 scenario. The classical RRT* and Bi-RRT* perform poorly in both success rate and path quality, as reflected by their zero-valued distributions. Overall, the boxplots further validate the effectiveness and robustness of the proposed method across different environments.

Based on the results presented above, it is evident that the proposed algorithm demonstrates significant improvement across both simulation maps. For the Simulation 1 map, the algorithms lacked goal point guidance and could not reach the goal configuration within the iteration limit, as seen from RRT* and Bi-RRT* having the success rates of 0% and 10%, respectively. According to the recorded data, these algorithms often exhausted all iterations without adequately exploring the joint space. The key point here is the high-dimensional space. For a 6-DOF manipulator, the number of possible joint configurations is extremely large, and random exploration alone becomes inefficient for traversing such the entire space. For the Simulation 2 map, this scenario requires the manipulator to pass through a narrow rectangular opening, which in 3D motion requires the manipulator to first fold inward and then extend forward. However, such coordinated motion is particularly difficult to achieve using sampling-based planners. If the algorithm only has the goal point guidance, it will most likely be stuck in front of the wall and waste a lot of iterations. Even with the assistance of repulsive function, the planner pushes the joint backward vertically but does not actually fold the manipulator in a way that is similar to human behavior. Under such conditions, an alternative strategy involving end-effector path planning followed by inverse kinematics may offer a more effective solution for identifying feasible joint configurations of the whole manipulators.

To further validate the theoretical analysis of expansion strategies, we conduct a comparative study using identical configurations, differing only in the expansion method by whether it is single-tree or dual-tree. The results, summarized in Table 4 and Table 5, highlight the practical efficiency gains of the proposed dual-tree approach.

In the Simulation 1 map, the dual-tree strategy demonstrates a substantial advantage in computational efficiency, reducing the average planning time from 17.66 s to 3.18 s while achieving comparable path lengths and maintaining a 100% success rate. This confirms the benefit of cooperative exploration in cluttered environments, where two trees can effectively divide the search space and converge more quickly.

In the Simulation 2 map, the performance gap narrows. Although the dual-tree approach still achieves full success, it exhibits longer average paths and moderately reduced computational gains compared to the single-tree variant. This observation aligns with a known limitation of bidirectional planners: difficulty in navigating through narrow passages, where synchronizing the growth of both trees becomes more challenging.

Despite this, the dual-tree expansion consistently avoids any computational overhead and often accelerates convergence in practice. These empirical results complement the theoretical analysis, confirming that the dual-tree strategy retains the O(NlogN) time complexity while improving planning efficiency across diverse environments.

However, one inherent limitation of the dual-tree approach lies in the quality of the connection point between the two trees. Since each tree optimizes its own path independently, the final path is formed by stitching two locally optimized subpaths. If the connection point does not lie on the globally optimal trajectory, the resulting path may deviate slightly from the global optimum. Nevertheless, this trade-off is often acceptable in practice given the significant gains in planning efficiency, as evidenced in both simulation scenarios.

In summary, the proposed GBAPF-RRT* algorithm is specifically designed for motion planning in high-dimensional joint spaces. It demonstrates strong performance in handling narrow passages and complex obstacle distributions. The proposed algorithm achieves shorter paths and faster runtimes while maintaining a high success rate, showing great potential for practical robotic applications.

## 6. Real World Experiment

### 6.1. Experiment Setup

**Figure 14:**
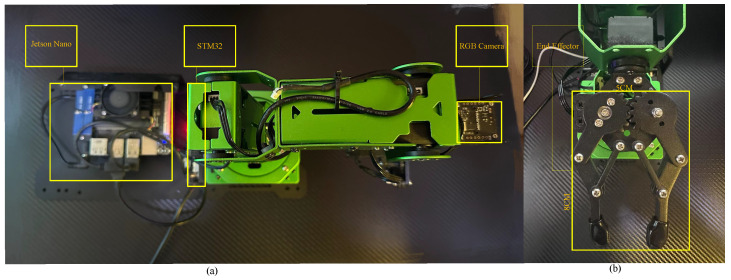
(**a**) Overview of the robotic manipulator platform. (**b**) Detailed view of the end-effector gripper, including dimensional annotations.

To evaluate the practical feasibility of the proposed algorithm for collision-free motion planning in real-world environments, a series of physical experiments were conducted. The experimental platform consists of a common commercially available 6-DOF robotic manipulator, characterized by sufficient kinematic flexibility, precise joint control, and a moderate payload capacity, making it suitable for validating high-dimensional planning strategies. The manipulator is equipped with a Jetson Nano onboard computer (NVIDIA Corporation, Santa Clara, CA, USA) running Ubuntu 18.04. Additionally, an RGB camera was originally mounted on the end-effector to localize target objects. However, the camera module was removed during the experiments in order to minimize the end-effector volume and improve clearance in confined spaces. The final size of the end-effector was approximately 8 cm × 5 cm × 5 cm. An illustration of the robotic platform is shown in Figure 14.

Initially, the experimental environment was constructed in MATLAB R2024a, where a simulated obstacle map was defined with dimensions corresponding to the real-world setup. The robot’s kinematic model strictly follows the DH parameters, as detailed in Table 1, which is a standard approach for modeling articulated manipulators. It should be noted that the DH-based model, as is typical, does not explicitly include the physical length of the end-effector gripper, which is treated as an external tool frame. This modeling approach ensures structural consistency while allowing effective simulation-based planning.

In terms of trajectory planning, results were first obtained through simulations. The proposed algorithm was used to generate a collision-free path, where each node in the RRT* tree corresponds to a target joint configuration. These configurations, consisting of six joint angles, were sequentially transmitted to the manipulator’s controller, which then executed them on the physical robot to ensure smooth and continuous motion. It is important to note that, in the MATLAB simulation, the end-effector was modeled as the final joint without accounting for the actual physical length of the gripper. This simplification led to a discrepancy between the simulated and real-world end-effector positions. To address this, the placement of obstacles in the physical environment was manually adjusted. This ensured consistent and reliable execution of the planned trajectories in the real-world setup.

### 6.2. Real-World Experiment 1: Narrow Vertical Gap

**Figure 15:**
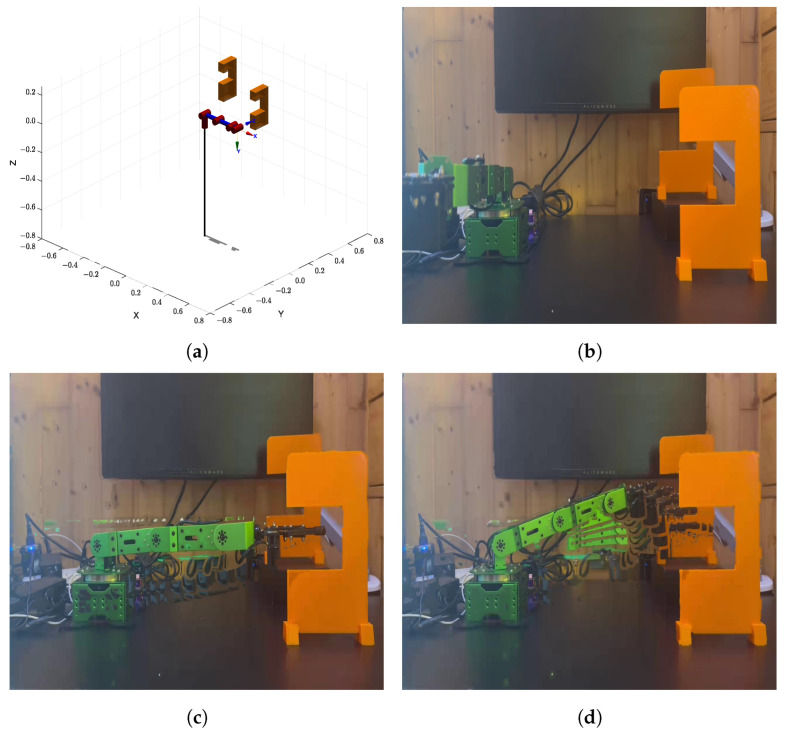
(**a**) Modeled environment 1 in MATLAB. (**b**–**d**) Sequential snapshots of the manipulator executing the planned trajectory in the real-world environment.

The first experiment was conducted in an environment featuring a narrow vertical gap with a fixed opening size of 8 cm × 6.5 cm, which posed a significant challenge by requiring the manipulator to precisely adjust its posture to navigate through the constrained space. As illustrated in Figure 15a shows the modeled environment in MATLAB, while Figure 15b–d sequentially present the actual trajectories executed by the manipulator in the real-world setting. The manipulator successfully followed the pre-planned path without any collisions. Over ten repeated trials, the algorithm consistently generated feasible trajectories that were accurately executed by the manipulator, achieving a 100% success rate. No notable deviations were observed between the planned and actual trajectories, highlighting the robustness of the proposed method in constrained environments.

### 6.3. Real-World Experiment 2: Rectangular Opening

**Figure 16:**
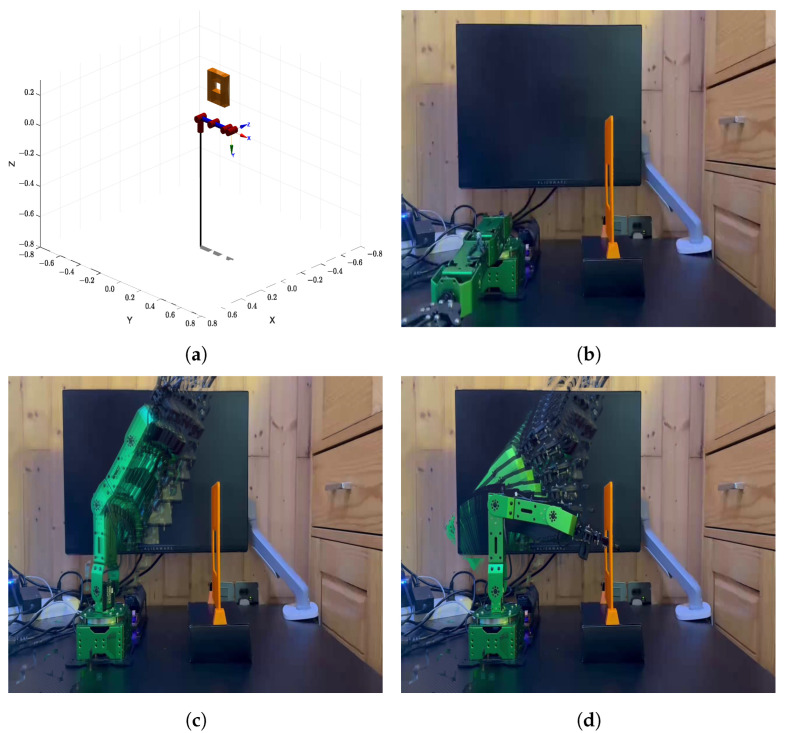
(**a**) Modeled environment 2 in MATLAB. (**b**–**d**) Sequential snapshots of the manipulator executing the planned trajectory in the real-world environment.

In the second scenario, the manipulator was required to pass through a narrow rectangular opening with dimensions of 8 cm × 6.5 cm. The environment map used in this experiment as shown in Figure 16a, while Figure 16b–d sequentially display the execution of the planned trajectory in the real-world setup. This scenario is designed to test the algorithm’s ability to generate feasible trajectories under tight spatial constraints. Because of slight differences between the model and the real manipulator, such as the length of the end-effector, the manipulator occasionally experienced minor tip collisions during execution.The randomness of the planning algorithm also led to different paths across trials. Even so, whenever a valid path was found, the manipulator followed it closely without major deviation. If no feasible path was found at first, the planner restarted automatically until success. Once a valid trajectory was available, the manipulator completed the task smoothly. These results show that the proposed method remains reliable and effective, even under real-world uncertainties and modeling differences.

## 7. Conclusions

This paper proposes an improved path-planning algorithm based on the joint space, termed GBAPF-RRT*. The proposed method introduces two important advantages. First, a goal-biased sampling mechanism is incorporated to improve the random sampling strategy of RRT*, effectively reducing the generation of invalid nodes and enhancing sampling efficiency. Second, an adaptive repulsion function, specifically designed for the characteristics of joint space, is developed to strengthen obstacle avoidance capabilities and improve overall path smoothness. These improvements collectively enable efficient, stable, and collision-free path planning. Lastly, extensive experiments were conducted on two representative environments. The results demonstrate that, compared with several mainstream RRT* variants, the proposed GBAPF-RRT* algorithm achieves significant improvements in both planning time and path length. In particular, it exhibits higher success rates and stronger robustness in environments with complex structures and constrained spaces.

## Figures and Tables

**Table 1:** Standard DH Parameters of the manipulator with 6-DOF.

Link *i*	$\theta_{i}$	$d_{i}$ [m]	$a_{i}$ [m]	$\alpha_{i}$ [rad]
1	0	0.05	0	$-\frac{\pi }{2}$
2	0	0	0.1294	0
3	0	0	0.1294	0
4	0	0	0.05	0
5	0	0	0	0
6	0	0	0	0

**Table 2:** Runtime and path length statistics on Map 1 (10 runs).

Algorithm	$l_{\min}$	$l_{\max}$	$l_{\mathrm{avg}}$	$t_{\min}$ (s)	$t_{\max}$ (s)	$t_{\mathrm{avg}}$ (s)	$n_{\mathrm{success}}$
RRT*	0	0	0	1780	1795	1788	0
Bi-RRT*	0	245	24	1670	3380	3184	1
G-Bi-RRT*	63	122	82	12	117	46	10
GBAPF-RRT*	20	34	28	1	8	4	10

**Table 3:** Runtime and path length statistics on Map 2 (10 runs).

Algorithm	$l_{\min}$	$l_{\max}$	$l_{\mathrm{avg}}$	$t_{\min}$ (s)	$t_{\max}$ (s)	$t_{\mathrm{avg}}$ (s)	$n_{\mathrm{success}}$
RRT*	0	0	0	1410	1430	1412	0
Bi-RRT*	0	300	30	1300	2700	2538	1
G-Bi-RRT*	95	203	135	28	156	81	10
GBAPF-RRT*	30	36	34	3	9	6	10

**Table 4:** Comparison of single-tree expansion with dual-tree expansion on GBAPF-RRT* in Simulation 1 map.

Algorithm	$l_{\min}$	$l_{\max}$	$l_{\mathrm{avg}}$	$t_{\min}$ (s)	$t_{\max}$ (s)	$t_{\mathrm{avg}}$ (s)	$n_{\mathrm{success}}$
Single Tree	24	32	28	12.13	25.10	17.66	5
Dual-Tree	26	30	27	2.01	4.40	3.18	5

**Table 5:** Comparison of single-tree expansion with dual-tree expansion on GBAPF-RRT* in Simulation 2 map.

Algorithm	$l_{\min}$	$l_{\max}$	$l_{\mathrm{avg}}$	$t_{\min}$ (s)	$t_{\max}$ (s)	$t_{\mathrm{avg}}$ (s)	$n_{\mathrm{success}}$
Single Tree	31	37	33	2.92	35.41	17.17	5
Dual-Tree	32	45	38	4.00	15.15	7.70	5

## Data Availability

The original datasets of this study are outlined within the article. For further inquiries, please contact the corresponding author.
